# A comparative analysis of transformed indolent lymphomas and de novo diffuse large B-cell lymphoma: a population-based cohort study

**DOI:** 10.1038/s41408-024-01194-5

**Published:** 2024-11-29

**Authors:** John L. Vaughn, Angela Ramdhanny, Malak Munir, Sravani Rimmalapudi, Narendranath Epperla

**Affiliations:** 1 Division of Hematology & Oncology, NYU Grossman Long Island School of Medicine, New York, NY USA; 2 Department of Medicine, Ain Shams University Faculty of Medicine, Cairo, Egypt; 3 Division of Hematology and Hematologic Malignancies, Huntsman Cancer Institute, University of Utah, Salt Lake City, UT USA

**Keywords:** Risk factors, Medical research

## Abstract

Histologic transformation (HT) of indolent non-Hodgkin lymphoma (iNHL) to diffuse large B-cell lymphoma (DLBCL) carries a poor prognosis. Using the Surveillance, Epidemiology, and End Results-17 database, we conducted a population-based study of adult patients with transformed follicular lymphoma (t-FL), marginal zone lymphoma (t-MZL), lymphoplasmacytic lymphoma/Waldenström macroglobulinemia (t-LPL/WM), and de novo DLBCL. Primary outcome was relative survival (RS), and secondary outcomes included overall survival (OS) and lymphoma-specific survival (LSS). Outcomes were modeled using flexible parametric survival models, while multivariable modeling was used to compare RS, OS, and LSS. The incidence of HT was highest in splenic MZL (SMZL, 6.78%) and lowest in extranodal MZL (EMZL, 1.62%). Median follow-up times were similar for patients with de novo DLBCL and transformed indolent lymphomas. The 5-year RS and OS were longer in de novo DLBCL compared to all other transformed iNHL subtypes (68 versus 59%, respectively). For t-FL, early transformation (within 2 years of diagnosis, Hazard ratio [HR] = 1.34) and prior treatment (HR = 1.89) were associated with inferior survival. This association was not observed in other transformed lymphoma subtypes. This is the first comparative study to show that the outcomes of t-LPL/WM were inferior compared to de novo DLBCL and highlights the need to incorporate early experimental therapies in patients with t-FL with early transformation and receipt of prior chemotherapy.

## Introduction

Histologic transformation (HT) from indolent non-Hodgkin lymphoma (iNHL) to aggressive lymphoma usually heralds a poor prognosis. For patients with B-cell lymphomas, this transformation is a pivotal event in the natural history and clinical course of the disease. Most indolent lymphomas have a long, protracted course for many years. However, a portion of patients experience HT to either diffuse large B-cell lymphoma (DLBCL) or a higher-grade morphology [1].

Although the rate of HT and outcomes vary among the different iNHL subtypes, they all portend inferior outcomes [1]. Previous studies have examined the outcomes of individual iNHL subtypes, including follicular lymphoma (FL), marginal zone lymphoma (MZL), and lymphoplasmacytic lymphoma/Waldenstrom macroglobulinemia (LPL/WM) [2–19], however, there is a paucity of data evaluating the outcomes of all transformed iNHLs with de novo DLBCL at a population level. Hence, we sought to estimate the risk of HT and survival in patients with transformed FL (t-FL), transformed MZL (t-MZL), and transformed LPL/WM (t-LPL/WM) using the Surveillance, Epidemiology, and End Results-17 (SEER-17) database. We hypothesize that transformed iNHLs have an inferior survival compared to patients with de novo DLBCL.

## Methods

### Study design and population

We conducted a population-based cohort study of patients with t-FL, t-MZL, t-LPL/WM, and de novo DLBCL using the SEER-17 database. The SEER-17 database covers an estimated 26.5% of the US population based on the 2020 US Census. We included adults (18–75 years at the time of their diagnosis) with histologically confirmed FL, MZL, or LPL/WM as their first malignant primary tumor diagnosed between 2010–2015. This time period was chosen to account for time to transformation and to allow for adequate follow-up. Patients who were diagnosed by autopsy or death certificate and those with primary central nervous system (CNS) lymphomas were excluded. We identified patients with HT by following patients from their diagnosis of iNHL to their subsequent diagnosis of DLBCL. All patients with HT had biopsy-proven rather than clinically suspected transformation. All pathology reports were reviewed by cancer registrars to ensure that all patients had histologically confirmed DLBCL prior to being reported to SEER.

We also included a separate cohort of patients with de novo DLBCL for comparison. This cohort included adults (18–89 years at the time of diagnosis) with histologically confirmed de novo DLBCL as their first malignant primary tumor between 2010–2020. Patients with primary CNS lymphomas and those who were diagnosed by autopsy or death certificate were excluded.

For all included patients, we identified patients using the SEER Lymphoid Neoplasm Recode 2021 Revision variable, which is based on International Classification of Disease (ICD) codes. We identified primary CNS lymphomas using the ICD for Oncology topology codes C700-701, C709-729, and C751-753. All patients included were followed through the end of 2020. Patients with concurrent low-grade lymphomas and DLBCL (“composite lymphomas”) were coded as DLBCL. Patients with grade 3 A and 3B FL were coded as having grade 3 FL and were not distinguished from each other.

### Ethics approval and consent to participate

The study was conducted in compliance with the Declaration of Helsinki. Given the nature of the study (population based study from a public repository), this study was IRB exempt. As this was a retrospective study, informed consent was waived.

### Outcomes and covariates

The primary outcome was relative survival (RS). Secondary outcomes were overall survival (OS) and lymphoma-specific survival (LSS). RS was defined as the ratio of all-cause survival to expected survival in a comparable group of individuals from the general population. We estimated expected survival by matching patients in our study to individuals in the general population by age, sex, year, and race using data provided by SEER. OS was defined as the probability of death from any cause following diagnosis of lymphoma. LSS was defined as the probability of survival when lymphoma was considered the only possible cause of death. We determined the cause of death using the SEER cause-specific death classification variable. The study covariates were age at diagnosis, year of diagnosis, sex, race (White, Black, and Other), Ann Arbor stage (stage I-II and stage III-IV), and presence of B symptoms. We modeled age and year as continuous variables using restricted cubic splines with three knots.

### Statistical analysis

We analyzed patient characteristics using descriptive statistics. We tested differences between categorical and continuous variables using Pearson’s chi-squared test and the Kruskal–Wallis test, respectively. We estimated the median follow-up time using the reverse Kaplan–Meier method. We modeled the study outcomes using flexible parametric survival models with six knots for the baseline cumulative hazard. We used multivariable modeling to compare RS, OS, and LSS between patients with transformed indolent lymphomas and de novo DLBCL by using HT as the key independent variable and adjusting for the study covariates. We modeled HT as a time-dependent variable using restricted cubic splines with three knots. We handled missing data using multiple imputations with chained equations. *P* values less than 0.05 were considered significant. Analyses were performed using Stata Basic Edition 18.0 (College Station, TX).

## Results

### Patient characteristics

There were 19,921 patients with iNHL diagnosed in the United States between 2000–2015. Among these, 11,934 were FL, 4070 were extranodal MZL (EMZL), 1,779 were nodal MZL (NMZL), 516 were splenic MZL (SMZL), and 1622 were LPL/WM. These patients were followed through 2020 to identify patients with HT. The median follow-up prior to HT was similar between lymphoma subtypes (Table 1). The cumulative incidence of HT was highest for SMZL (6.78%), followed by FL (5.55%), NMZL (4.05%), LPL/WM (2.22%), and EMZL (1.62%).

**Table 1:** Cumulative incidence of histologic transformation by lymphoma subtype.

	Patients diagnosed between 2010–2015, *n*	Transformed cases between 2010–2020, *n*	Median follow-up among survivors, y (95% CI)	Cumulative incidence of HT, %
FL	11934	662	7.75 (7.67–7.83)	5.55
EMZL	4070	66	7.58 (7.50–7.67)	1.62
NMZL	1779	72	7.83 (7.67–8.08)	4.05
SMZL	516	35	7.67 (7.42–8.08)	6.78
LPL/WM	1622	36	7.67 (7.50–7.83)	2.22

*HT* histologic transformation, *FL* follicular lymphoma, *EMZL* extranodal marginal zone lymphoma, *NMZL* nodal marginal zone lymphoma, *SMZL* splenic marginal zone lymphoma, *LPL/WM* lymphoplasmacytic lymphoma/Waldenstrom’s macroglobulinemia.

Baseline clinicodemographic characteristics for patients with HT were compared to those with de novo DLBCL, as shown in Table 2. Compared to patients with de novo DLBCL, patients with transformed lymphoma were younger at diagnosis (median age 64 vs 66 years, *P* = 0.001), were more likely to be White (87 vs 81%, *P* < 0.001), were more likely to have advanced disease (stage 3–4, 58 vs 54%, *P* < 0.001), and were less likely to have documented B symptoms (18 vs 29%, *P* < 0.001). Median follow-up times were similar for patients with de novo DLBCL and transformed indolent lymphomas (Table S1).

**Table 2:** Patient characteristics for de novo DLBCL and transformed lymphomas.

	de novo DLBCL	Transformed	*p* value
	*N* = 51,215	*N* = 871	
Age at diagnosis, median (IQR)	66 (55–75)	64 (57–71)	0.001
Age category			<0.001
18–44	6271 (12%)	26 (3%)	
45–54	6516 (13%)	142 (16%)	
55–64	11,206 (22%)	270 (31%)	
65–74	13,168 (26%)	322 (37%)	
75–89	14,054 (27%)	111 (13%)	
Sex			0.27
Female	22,552 (44%)	400 (46%)	
Male	28,663 (56%)	471 (54%)	
Race			<0.001
White	41,415 (81%)	762 (87%)	
Black	3919 (8%)	39 (4%)	
Other	5375 (10%)	66 (8%)	
Missing	506 (1%)	4 (<1%)	
Ann Arbor stage			<0.001
Stage I-II	20,714 (40%)	274 (31%)	
Stage III_IV	27,594 (54%)	508 (58%)	
Missing	2907 (6%)	89 (10%)	
B symptoms			<0.001
No B symptoms	28,015 (55%)	488 (56%)	
B symptoms	14,618 (29%)	161 (18%)	
Missing	8582 (17%)	222 (25%)	

*DLBCL* diffuse large B-cell lymphoma.

### Survival analysis

The unadjusted five-year RS rates were highest for de novo DLBCL at 68%, followed by t-EMZL (61%), t-FL (58%), t-NMZL (56%), t-SMZL (54%), and T-LPL/WM (36%) (Table 3). A similar pattern was observed for OS and LSS, with de novo DLBCL having the highest survival (5-year OS and LSS of 59 and 68%, respectively) and t-LPL/WM (5-year OS and LSS of 33 and 45%, respectively) having the lowest survival. We also compared the outcomes of patients with de novo DLBCL to patients with HT using multivariable analysis. On multivariable analysis after adjusting for age, sex, race, stage, and presence of B symptoms, patients with t-FL (hazard ratio [HR] 1.42, 95% CI, 1.24–16.2; *P* < 0.001) and t-LPL/WM (HR 1.65, 95% CI, 1.02–2.67; *P* = 0.04) had significantly higher excess hazard rates compared to patients with de novo DLBCL (Table 4).

**Table 3:** Survival for de novo DLBCL and transformed lymphomas.

	5-year RS (95% CI)	5-year OS (95% CI)	Median OS, y (95% CI)	5-year LSS (95% CI)
De novo DLBCL	0.68 (0.67–0.68)	0.59 (0.58–0.59)	9.25 (8.91–9.59)	0.68 (0.67–0.68)
t-FL	0.58 (0.53–0.62)	0.52 (0.48–0.56)	5.85 (4.33–7.37)	0.58 (0.54–0.62)
t-EMZL	0.61 (0.46–0.73)	0.54 (0.41–0.66)	6.45 (1.26–11.64)	0.68 (0.54–0.79)
t-NMZL	0.56 (0.41–0.68)	0.51 (0.38–0.62)	5.35 (1.45–9.25)	0.57 (0.44–0.68)
t-SMZL	0.54 (0.34–0.71)	0.51 (0.33–0.67)	5.29 (0–10.61)	0.64 (0.43–0.79)
t-LPL/WM	0.36 (0.19–0.53)	0.33 (0.18–0.48)	2.06 (0.40–3.72)	0.45 (0.27–0.61)

*DLBCL* diffuse large B-cell lymphoma, *t-FL* transformed follicular lymphoma, *t-EMZL* transformed extranodal marginal zone lymphoma, *t-NMZL* transformed nodal marginal zone lymphoma, *t-SMZL* transformed splenic marginal zone lymphoma, *t-LPL/WM* transformed lymphoplasmacytic lymphoma/Waldenstrom’s macroglobulinemia, *RS* relative survival, *OS* overall survival, *LSS* lymphoma-specific survival.

**Table 4:** Flexible parametric survival models comparing de novo DLBCL and transformed lymphomas.

	RS		OS		LSS	
De novo DLBCL	HR (95% CI)	*P* value	HR (95% CI)	*P* value	HR (95% CI)	*P* value
Unadjusted	Reference		Reference		Reference	
Adjusted	Reference		Reference		Reference	
t-FL						
Unadjusted	1.26 (1.10–1.44)	0.001	1.12 (0.99–1.26)	0.07	1.23 (1.08–1.41)	0.002
Adjusted^a^	1.42 (1.24–1.62)	<0.001	1.35 (1.20–1.53)	<0.001	1.48 (1.30–1.68)	<0.001
t-EMZL						
Unadjusted	1.01 (0.64–1.59)	0.96	0.97 (0.66–1.43)	0.89	0.78 (0.48–1.27)	0.32
Adjusted^a^	0.95 (0.61–1.49)	0.83	0.96 (0.65–1.40)	0.82	0.76 (0.47–1.23)	0.27
t-NMZL						
Unadjusted	1.06 (0.70–1.60)	0.78	0.97 (0.68–1.40)	0.89	1.03 (0.69–1.53)	0.90
Adjusted^a^	1.24 (0.83–1.85)	0.30	1.20 (0.84–1.73)	0.32	1.25 (0.84–1.86)	0.28
t-SMZL						
Unadjusted	0.98 (0.55–1.74)	0.94	0.90 (0.53–1.51)	0.68	0.72 (0.38–1.37)	0.32
Adjusted^a^	0.87 (0.49–1.56)	0.64	0.84 (0.50–1.41)	0.32	0.67 (0.35–1.27)	0.22
t-LPL/WM						
Unadjusted	1.46 (0.89–2.40)	0.14	1.35 (0.86–2.11)	0.19	1.17 (0.69–1.97)	0.56
Adjusted^a^	1.65 (1.02–2.67)	0.04	1.47 (0.94–2.29)	0.09	1.32 (0.78–2.22)	0.30

*DLBCL* diffuse large B-cell lymphoma, *t-FL* transformed follicular lymphoma, *t-EMZL* transformed extranodal marginal zone lymphoma, *t-NMZL* transformed nodal marginal zone lymphoma, *t-SMZL* transformed splenic marginal zone lymphoma, *t-LPL/WM* transformed lymphoplasmacytic lymphoma/Waldenstrom’s macroglobulinemia, *RS* relative survival, *OS* overall survival, *LSS* lymphoma-specific survival.

^a^Models were adjusted for age, sex, race, stage, and presence of B symptoms.

### Early transformation and prior therapy

We next examined the associations between early HT and receipt of prior chemotherapy on survival. The frequency of early transformation, defined as HT within 2 years of iNHL diagnosis, varied according to the subtype of lymphoma. Patients with FL had the highest frequency of early transformation (*n* = 311, 47%), followed by t-LPL/WM (*n* = 15, 42%) and t-MZL (all subtypes combined, *n* = 62, 36%). Early transformation was associated with inferior survival for patients with t-FL but not with other lymphoma subtypes (HR 1.34; 95% CI, 1.03–1.74; *P* = 0.03) (Table S2).

A documented history of chemotherapy prior to HT was highest for t-FL (*n* = 333, 50%), followed by t-LPL/WM (*n* = 17, 47%) and t-MZL (*n* = 65, 38%). Receipt of prior chemotherapy was associated with inferior survival for t-FL but not for other lymphoma subtypes (HR 1.89; 95% CI, 1.45–2.48; *P* < 0.001) (Table S3). Compared to chemotherapy, fewer patients received radiation therapy. The proportion of patients receiving radiation therapy prior to HT was highest for t-MZL (43/173, 20%), followed by t-FL (68/594, 11%) and t-LPL/WM (3/36, 8%). There was no association between receipt of prior RT and survival for any lymphoma subtype (Table S4)

## Discussion

In this comprehensive US population-level comparative analysis of patients with transformed iNHL and de novo DLBCL, we report several important observations. First, the incidence of HT was highest in SMZL, followed by FL, and lowest in EMZL. Second, 5-year RS, OS, and LSS was better in patients with de novo DLBCL compared to transformed iNHL subtypes. Among the patients with iNHL, t-LPL/WM had the worse survival rates relative to the other subtypes. Third, early HT and receipt of prior chemotherapy was associated with inferior survival for patients with t-FL but not other iNHL subtypes. Our findings add to the growing body of literature on the epidemiology of HT of iNHLs and will serve as a guide to explore further research in tailoring the management based on the transformed iNHL subtype and the timing of HT.

Notably, for t-FL, our results are lower than those reported in other studies conducted after the introduction of rituximab, with studies reporting a cumulative incidence of HT in FL of 10.7% at 5 years, between 4.1 to 15.3% at 6 years, and 7.7 to 8% at 10 years [2–5, 7]. However, in our analysis, we report a lower rate of HT (5.5%) at a median follow-up of 7.75 years, a finding closer to that recently published study by Florindez et al. [6] These discrepancies may be attributed to variations in population characteristics, geographic factors influencing treatment decisions, study design (including rituximab treatment rates), and assessment methodologies. The cumulative incidence of HT in MZL at 10 years ranged from 2.95 to 8.4% across various studies, with SMZL having the highest incidence and EMZL with the lowest rates of HT [6, 13, 14, 17, 19], which is in line with our study. Moreover, the rate of HT in LPL/WM in our study was similar to the previously reported 2.4% at both 5 and 10 years [16, 18]. Our analysis, which reports a 2.2% cumulative incidence over a median of 7.67 years, bridges these two time points. The discrepancies among these findings could be attributed to several factors, notably the rarity of WM/LPL, the underlying treatment strategies, as well as the power of a population-level analysis compared to smaller cohorts.

The differences in the incidence of HT across different lymphoma subtypes may be attributed to distinct molecular mechanisms that drive the transformation in each. For example, in FL, genome-wide studies have provided insight into the molecular alterations that contribute to HT. T-FL has been shown to harbor a high mutational burden, particularly due to increased copy number aberrations, mutational structural rearrangements, and somatic hypermutation of target genes, thus increasing genomic complexity and instability [20, 21]. There is emerging data regarding the clinicopathological and molecular characteristics of MZL and WM leading to HT. Studies have shown that the presence of monoclonal protein [22], high Ki-67% [23], and lack of achievement of complete response to first-line treatment were associated with an increased risk of transformation of MZL [17]. Additionally, comprehensive molecular analyses identified NF-kB signaling genes (TNFAIP3 and KMT2D), NOTCH/2 pathway genes, KLF2, and TP53 as the most commonly altered genes in t-SMZL, with KLF2 and complex chromosomal structures being associated with inferior survival [24–26]. In WM, the presence of wild-type MYD88 has been shown to confer a higher risk of HT [18, 27, 28] associated with carrying a higher risk of mutation in several genes that contribute to NF-kB signaling, similar to the genetic profile of de novo DLBCL [29, 30].

In contrast to two non-population level studies reporting no difference in overall survival in transformed versus de novo DLBCL [15, 31], our analysis showed inferior survival in transformed iNHL compared to de novo DLBCL. Our adjusted analysis supports the findings of recent studies showing a significantly higher risk of mortality in patients with t-FL [6, 12] compared to de novo DLBCL. However, in contrast to the recent study by Florindez et al. [6], this association was not statistically significant in any of the t-MZL subtypes. To our knowledge, we are the first to report an increased risk of mortality with t-LPL/WM compared to de novo DLBCL. Although the exact reasons are unclear for this trend, the poorer prognosis associated with t-FL and t-LPL/WM may be driven by the presence of high-risk molecular features that drive transformation, in addition to the influence of prior therapy.

We found that patients with FL had the highest frequency of early HT, with 47% of patients experiencing HT within 2 years of initial diagnosis, followed by t-LPL/WM (42%) and t-MZL (36%). We noted that the occurrence of early HT was associated with inferior OS in patients with t-FL, with a 34% increase in risk of mortality compared to late HT, which is in line with the prior studies [5–7]. However, we did not see any association between the timing of HT on outcomes in t-MZL and t-LPL/WM. While the impact of timing of HT on outcomes in t-MZL has been shown in prior work [6], this is the first study to evaluate the impact of timing of HT on outcomes in patients with t-LPL/WM. This is important information that can be useful while counseling patients.

Receipt of chemotherapy prior to HT was highest for t-FL, followed by t-LPL/WM and t-MZL. In line with published data prior treatment was associated with inferior survival for t-FL [6, 8, 9], however this finding was not significant with other subtypes. One possible explanation for this observation in FL is that prior exposure to chemotherapy may select resistant clones that contribute to the development of transformation and treatment refractoriness [1, 32].

Our study is limited by a lack of certain clinicopathologic information at the patient level, such as International Prognostic Index scores, cytogenetic abnormalities, and molecular alterations. These data are not consistently reported to cancer registries in the United States and are not included in the SEER database. While there was information pertaining to the receipt of prior systemic therapy, the exact details of the treatment regimen, including prior anthracycline exposure, are not available in the SEER cancer registry. Hence, we could not match the two groups (de novo DLCBL and transformed lymphoma) for the type of systemic therapy and number of lines of therapy.

In conclusion, our findings provide a comprehensive insight into the incidence of transformation and prognosis of transformed iNHLs. To our knowledge, this is the first comparative study to show that the outcomes of patients with t-LPL/WM were inferior compared to de novo DLBCL. We also identified different rates of transformation for MZL subtypes, with the highest incidence in SMZL. This emphasizes the need to re-biopsy when SMZL patients present with the progression of the disease to rule out HT. We found that patients with transformed iNHLs have poor survival compared to de novo DLBCL, which is important for counseling patients in the clinic. Additionally, the poor prognosis associated with early HT and receipt of prior chemotherapy in patients with t-FL underscores the need for early institution of experimental therapies in this high-risk subgroup.

## Supplementary information

Supplementary Appendix

## Data Availability

This study used publicly available data, which can be accessed through the Surveillance, Epidemiology, and End Results (SEER)-17 database.
