# Supplementary material for: A comparative analysis of transformed indolent lymphomas and de novo diffuse large B-cell lymphoma: a population-based cohort study
Source: Blood Cancer J. 2024 Nov 29;14(1):212. doi: 10.1038/s41408-024-01194-5 (PMC11604794; doi:10.1038/s41408-024-01194-5)
Supplement: Supplementary file 1 — Supplementary Appendix [file 41408_2024_1194_MOESM1_ESM.docx]

**SUPPLEMENTAL APPENDIX**

**Table of Contents** 1

**Table S1. Median follow-up times for *de novo* DLBCL and transformed indolent lymphomas** 2

**Table S2. Relative survival according to early versus late transformation** 3

**Table S3. Relative survival according to receipt of prior chemotherapy** 4

**Table S4. Relative survival according to receipt of prior radiation therapy** 5

**Table S1. Median follow-up times for *de novo* DLBCL and transformed indolent lymphomas**

| **Disease** | **Median follow-up, years (95% CI)** |
| --- | --- |
| *De novo* DLBCL | 4.92 (4.83-5.00) |
| t-FL | 4.58 (4.17-5.00) |
| t-EMZL | 4.50 (2.75-5.92) |
| t-NMZL | 4.92 (3.00-5.83) |
| t-SMZL | 4.08 (2.67-6.5) |
| t-LPL/WM | 5.00 (3.83-6.83) |

**Table S2. Relative survival according to early versus late transformation**

|  | **Early Transformation [n (%)]** | **HR (95% CI)*** | **P-value** |
| --- | --- | --- | --- |
| t-FL | 311/662 (47%) | 1.34 (1.03-1.74) | 0.03 |
| t-MZL (all subtypes) | 62/170 (36%) | 1.05 (0.61-1.80) | 0.87 |
| t-LPL/WM | 15/36 (42%) | 0.54 (0.20-1.43) | 0.21 |

*Patients with late transformation were used as the reference.

**Table S3. Relative survival according to receipt of prior chemotherapy**

|  | **Prior Chemotherapy [n (%)]** | **HR (95% CI)*** | **P-value** |
| --- | --- | --- | --- |
| t-FL | 333/662 (50%) | 1.89 (1.45-2.48) | <0.001 |
| t-MZL (all subtypes) | 65/170 (38%) | 1.12 (0.65-1.92) | 0.69 |
| t-LPL/WM | 17/36 (47%) | 0.62 (0.23-1.63) | 0.33 |

*Patients without a documented history of chemotherapy were used as the reference.

**Table S4. Relative survival according to receipt of radiation therapy**

|  | **Prior Radiation Therapy [n (%)]** | **HR (95% CI)*** | **P-value** |
| --- | --- | --- | --- |
| t-FL | 68/594 (11%) | 0.78 (0.49-1.25) | 0.30 |
| t-MZL (all subtypes) | 43/173 (20%) | 1.04 (0.53-2.05) | 0.90 |
| t-LPL/WM | 3/36 (8%) | ** | ** |

*Patients without a documented history of radiation therapy were used as the reference.

**Unable to estimate due to the low numbers.
